# Continuity of midwifery care and gestational weight gain in obese women: a randomised controlled trial

**DOI:** 10.1186/1471-2458-11-174

**Published:** 2011-03-22

**Authors:** Cate Nagle, Helen Skouteris, Anne Hotchin, Lauren Bruce, Denise Patterson, Glyn Teale

**Affiliations:** 1 School of Nursing and Midwifery, Deakin University, Geelong Waterfront campus, 1 Gheringhap St, Geelong Victoria, 3217, Australia; 2 School of Psychology, Burwood campus, 221 Burwood Hwy, Burwood Victoria, 3125, Australia; 3 Department of Obstetrics and Gynaecology, Barwon Health, Ryrie St, Geelong Victoria, 3215, Australia; 4 Women's and Children's Program, Box Hill Hospital, Arnold St, Box Hill, Victoria, 3128, Australia; 5 Department of Obstetrics and Gynaecology, Goulburn Valley Health, Graham St, Shepparton, Victoria, 3630, Australia

## Abstract

**Background:**

The increased prevalence of obesity in pregnant women in Australia and other developed countries is a significant public health concern. Obese women are at increased risk of serious perinatal complications and guidelines recommend weight gain restriction and additional care. There is limited evidence to support the effectiveness of dietary and physical activity lifestyle interventions in preventing adverse perinatal outcomes and new strategies need to be evaluated. The primary aim of this project is to evaluate the effect of continuity of midwifery care on restricting gestational weight gain in obese women to the recommended range. The secondary aims of the study are to assess the impact of continuity of midwifery care on: women's experience of pregnancy care; women's satisfaction with care and a range of psychological factors.

**Methods/Design:**

A two arm randomised controlled trial (RCT) will be conducted with primigravid women recruited from maternity services in Victoria, Australia. Participants will be primigravid women, with a BMI≥30 who are less than 17 weeks gestation. Women allocated to the intervention arm will be cared for in a midwifery continuity of care model and receive an informational leaflet on managing weight gain in pregnancy. Women allocated to the control group will receive routine care in addition to the same informational leaflet. Weight gain during pregnancy, standards of care, medical and obstetric information will be extracted from medical records. Data collected at recruitment (self administered survey) and at 36 weeks by postal survey will include socio-demographic information and the use of validated scales to measure secondary outcomes.

**Discussion:**

Continuity of midwifery care models are well aligned with current Victorian, Australian and many international government policies on maternity care. Increasingly, midwifery continuity models of care are being introduced in low risk maternity care, and information on their application in high risk populations is required. There is an identified need to trial alternative antenatal interventions to reduce perinatal risk factors for women who are obese and the findings from this project may have application in other maternity services. In addition this study will inform a larger trial that will focus on birth and postnatal outcomes.

**Trial Registration:**

**Australian New Zealand Clinical Trials Registry **ACTRN12610001078044.

## Background

Obesity in pregnancy has increased to epidemic proportions in developed countries; it is associated with adverse outcomes for both mother and child [1] and is one of the most commonly occurring risk factors in maternity care [2]. Recent estimates suggest that approximately 35% of pregnant women in Australia have a body mass index (BMI) of greater than 25 kg/m^2 ^[3]. Maternal obesity is a well recognised perinatal risk factor [4] and it is associated with sub-fertility in women and an increased risk of miscarriage and stillbirth [5]. Maternal risks for hypertensive disorders [3,6-8], gestational diabetes [3,6,9] thrombo-embolism [8], haemorrhage, infections [8] and death [10] are increased in obese women and there are attendant risks and resourcing issues associated with obesity and childbirth. Compared to women who have a BMI between 20.1-25, obese women are more likely to experience: increased rates of induction of labour and a failed induction; anaesthetic difficulties [11-13]; a caesarean section [3,14-16]; a stay in hospital of more than five days [3] and obese women require specific equipment for accurate monitoring and safe maternity care [2]. The risks of adverse outcomes for fetuses and neonates are also increased. Maternal obesity is associated with fetal macrosomia, birth defects, prematurity, higher rates of admission to neonatal intensive care environments [3,12,17] and perinatal death [6,12,17,18]. The impact of obesity is pervasive with maternal obesity also being associated with postnatal effects of lower breastfeeding rates [19] and longer term with obesity in childhood [20]. Higher birth weight babies are associated with adolescent obesity [21] and are more likely to grow into obese adults [20].

In 2009 the American Institute of Medicine (IOM) published revised guidelines on how much weight a woman should gain during pregnancy and highlighted the importance of intervention in pregnancy to prevent *both *postpartum weight retention and childhood obesity [22]. The IOM recommends that women who are obese (BMI of 30 kg/m^2 ^and above) should gain approximately 5 to 9 kg. Excessive gestational weight gain is defined as weight gain above this recommended guideline. A recent systematic review of studies designed to prevent excessive gestational weight gain [23] revealed that, across seven studies, 63-74% of obese women gained more than 9 kg during their pregnancy and therefore exceeded the IOM guidelines for weight gain. Indeed, the adherence to recommended weight gain guidelines appears to be significantly lower for obese, as opposed to healthy weight, pregnant women [24]. There is limited evidence to support the effectiveness of dietary and physical activity lifestyle interventions in preventing adverse perinatal outcomes. In a systematic review of nine trials involving 743 women, Dodd and colleagues [25] concluded that the effectiveness of antenatal lifestyle interventions remain unclear. This is of significant concern and forms the rationale for trialling a different approach to preventing excessive weight gain in obese women.

Maternity reform at both federal [26] and state [27] levels of government promote the benefits of continuity of care and the expanded role of midwives working collaboratively in multidisciplinary teams. Continuity of care can be defined as care that is provided by the same clinician or small group of clinicians throughout pregnancy, birth and the postnatal period [28,29] and is often used in the context of models of midwifery care. There is evidence from a systematic review involving 11 trials and 12,276 women [30] that supports the beneficial effects of continuity of midwifery care on improving selected outcomes for women with no evidence of adverse outcomes. Hatem et al. propose that the underpinning philosophy of midwife-led care is normality and that caution needs to be exercised where medical complications exist and care needs a multidisciplinary approach [30].

Continuity of midwifery care can empower women and promote participation in their care [29] improve the sense of control they perceive [29,31], and improve satisfaction with care [29,31,32]. However, the impact of continuity of midwifery care as an intervention to improve outcomes for women who are obese has not been explored. It is important that both clinicians and obese women are aware of the need to limit gestational weight gain and participate in additional care, such as additional monitoring, in order to minimise the risk of maternal and fetal complications [2].

Evidence-based care should involve women being actively involved in their care, being provided with accurate information on risks and management in a sensitive manner [2]. In the absence of evidence based Australian guidelines, it is not surprising that currently there is significant variation in the quality and quantity of information that obese women receive in pregnancy. There is a particular science involved in the development of consumer health information where the topics are of a sensitive nature [33,34] in order to assist women to make informed choices. The need to measure continuity of midwifery care as an intervention for obese women is timely with the emergence of more midwifery led models in Australia. Evaluating the effect of a resource that is based on the latest evidence based guidelines is also identified as a significant need in clinical practice.

We plan to evaluate the impact of midwifery continuity of care on restricting gestational weight gain in obese women to the recommended range. Secondary aims of the study are to assess the impact of midwifery continuity of care on women's experience of and satisfaction with care and on a range of psychological factors. This paper describes the trial protocol in detail.

## Methods

This study employs a two arm unblinded randomised controlled trial (RCT) where pregnant women are allocated to either the intervention or control group. The study will be conducted and reported in line with CONSORT recommendations (Additional file 1.)

### Aims

This project has one primary aim: to measure the impact of continuity of midwifery care compared to routine care on restricting excessive gestational weight gain in obese women. Secondary aims are to measure the impact of continuity of midwifery care compared to routine care on obese women's experience of pregnancy care, women's satisfaction with care and selected psychological factors.

### Participants

The participants will be 214 primigravid women who attend one of the recruiting maternity services for pregnancy care (Eastern Health, Barwon Health and Goulburn Valley Health) during the recruitment period and at booking have a BMI≥30 and are less than 17 weeks gestation [8]. Women will be excluded from participating in the study if they: Are unable to give informed consent in English; have a multiple pregnancy; are currently experiencing vaginal bleeding; have a severe medical condition that prevents them from being randomised to a continuity of midwifery care model or have already commenced in a shared-care model of care with their General Practitioner.

### Recruitment strategies

Information on this study will be posted to all women with their initial appointment. Following the calculation of their BMI at the booking in visit, women eligible to participate will be provided with verbal and written information about this project by the attending doctor or midwife or a research assistant (RA) and invited to participate. Women will be given time to ask questions and will provide written informed consent prior to randomisation.

### Randomisation Procedure

Block randomisation will be undertaken using a computer generated randomisation sequence for each recruitment site. Following the recruitment of each woman, an opaque envelope will be selected in sequential order by the attending clinician and the contents will indicate allocation to the intervention group or the control group.

Women in the intervention group will receive 'continuity of care' defined as seeing the same midwife or small team of midwives for pregnancy care visits.

Women in the control group will receive usual clinical care. This may involve a care in one of any number of models of care available at the maternity service. Within these models of care, women see a variety of clinicians and are likely to see the same midwife at pregnancy care consultations only by chance.

All participants will receive an evidence based informational leaflet developed by the Nutrition and Dietetics Department at Goulburn Valley Health (Additional file 2). This will be modified at other sites to badge the relevant health service logo.

### Blinding

Given the nature of the intervention, blinding of researchers is not practical in this study, however a person independent of the study will keep the master list of all randomised participants and will audit the integrity of the randomisation prior to analysis.

Participating women will provide written consent prior to randomisation. Attrition bias will be minimised by all participants receiving an informational resource.

### Primary outcome and measures

#### Gestational weight gain

The primary outcome is the proportion of women with a gestational weight gain within IOM guidelines. A medical record review will compare the last weight recorded in pregnancy (taken at the maternity service at 36 weeks onwards and as close as practical to birth) with the woman's booking-in weight (taken at the maternity service when the woman's BMI is calculated).

### Secondary outcomes and measures

#### Women's experience of care

Provision of care in line with the standards within the UK guidelines [2] will be assessed by a review of medical records. Standards include:

Evidence of commencement of folic acid supplementation

Evidence of commencement of Vitamin D supplementation

Record of BMI recorded

Evidence of anaesthetic review if BMI≥40

Evidence of thromboprophylaxis ordered antenatally

Evidence of Glucose Tolerance Testing in pregnancy

Evidence of indications for Induction of Labour

#### Women's satisfaction with care

Validated scales used in large Victorian pregnancy cross-sectional studies [28,32] will be included to measure women's satisfaction with care, engagement and sense of control. Items use 7 point scales with extreme values verbally described (1 = disagree strongly, 7 = agree strongly). Global questions summarise the concept on balance and open ended questions provided an opportunity for additional comments.

#### Psychological factors during pregnancy

Factors such as readiness to change behaviour [35], anxiety [36], depression [37,38] and body image [39,40] will be assessed at baseline and at 36 weeks gestation.

##### Readiness to Change Questionnaire

This researcher designed measure modifies an existing scale [35] to the weight loss context. There are 12 items which are rated on a five point scale from Strongly agree to Strongly disagree. The items refer to an individual's *readiness, importance *and *confidence *in adopting healthy lifestyle changes during pregnancy, including subjective ratings of the healthiness of current eating and physical activity behaviours.

##### Anxiety

Anxiety will be measured using the short version of the Speilberger State-Trait Anxiety Inventory (State). Responses to six statements include 'Not at all', 'Somewhat', 'Moderately' and 'Very much' (alpha0.82)[36].

##### Depression

Depression will be measured using the Edinburgh Postnatal Depression Scale, which has been validated to use in pregnancy [37]. Women will be classified as probably clinically depressed using a cut-off score of ≥ 13, providing a sensitivity of 100% and specificity between 87 and 95.7% [38].

##### Body Attitudes Questionnaire

Two subscales of the Body Attitudes Questionnaire [39] which relate to pregnant women will be used to assess Body Image: Attractiveness and Salience of Weight and Shape. Responses range from *definitely disagree *to *definitely agree *for each of the 10 items; higher scores indicate greater amounts of each construct.

##### Pregnancy Figure Rating Scale

The Pregnancy Figure Rating Scale was developed to evaluate body dissatisfaction amongst pregnant women [40] and provides ratings of three body parts: bust, stomach and buttocks along a scale from 1 to 10 represented by figures increasing in size for each respective body part. Women rate the figures according to their *current *and *ideal *size for each body part. Body dissatisfaction is assessed using the discrepancy between current and ideal ratings for each body part [40].

Socio-demographic data such as age, ethnicity, education level and socio-economic status will be obtained in the first questionnaire completed at recruitment.

### Power, sample size and retention

A difference between groups of 20% is deemed clinically significant. The proportion of obese women gaining weight in excess of evidence based guidelines is approximately 65% [23]. It is estimated that 107 women per study arm (total of 214 participants) is required in order to detect a difference of 20% between groups with 80% power at a 0.05 level of significance [41]. Based on current booking numbers and the prevalence of obesity at the recruitment sites, approximately 74 women will be eligible to participate each month. To accommodate an anticipated 77% participation rate [42] and a 15% attrition rate [42], the required sample will be recruited in four and a half months.

### Data Collection

Two questionnaires, one completed at recruitment and the second posted at 36 weeks will provide the self reported measures and socio-demographic data. An audit of medical records will collect data on weight gain, standards of care, number of clinicians providing care, medical complications and obstetric details.

### Ethics

The study has been approved by the Human Research Ethics Committees of Eastern Health, Barwon Health and Goulburn Valley Health, Victoria Australia and endorsed by Deakin University.

### Analyses

Analyses will be undertaken using STATA [43] and comparability of baseline characteristics will be assessed for participants in both groups. The intervention group will be compared to the control group by intention to treat analysis. Proportions of women restricting weight gain to recommended levels will be compared using chi-square tests and odds ratios. Comparison of means will be undertaken for continuous variables using t-tests where data are normally distributed, otherwise medians will be compared using Mann-Whitney U tests. Ranked or Likert scales will be analysed using cumulative odds ratios and 95% confidence intervals will be reported. Where differences in baseline characteristics exist that may be associated with outcomes, additional multivariate analysis will be performed.

## Discussion

Continuity of midwifery care models usually involve the care of women at low risk of complications. There is an identified need to trial alternative antenatal interventions and the findings of this project will contribute evidence to improving care for obese women. The application of continuity to the midwifery care of women with a BMI≥30 needs rigorous evaluation as these women are at increased risk of complications and have numerous interactions with maternity care clinicians so there is the potential for fragmented care, miscommunication and confusion resulting from information being presented from a variety of sources. Continuity of midwifery care is increasingly being introduced as a model for low risk maternity care and before this model can be recommended to the care of women whose BMI is ≥30, safety and cost-effectiveness need to be evaluated. The findings from this study will inform a larger trial that will focus on birth and postnatal outcomes.

## Competing interests

The authors declare that they have no competing interests.

## Authors' contributions

CN and HS are chief investigators and have joint overall responsibility for the trial. AH conceived the study which was developed by CN and HS. CN, HS, AH, GT and DP wrote the initial grant application. LB, CN and HS drafted the initial questionnaires. CN, HS, AH, LB, GT and DP submitted ethics applications. CN, HS and LB drafted the trial protocol manuscript. All authors reviewed and approved the final manuscript.

## Pre-publication history

The pre-publication history for this paper can be accessed here:

http://www.biomedcentral.com/1471-2458/11/174/prepub

## Supplementary Material

Additional file 1 **CONSORT flow diagram**. This flow diagram depicts the adherence of this study to CONSORT requirements Click here for file

Additional file 2 **Informational leaflet**. This is the information leaflet on healthy weight gain that will be provided to women in both aims of this study. Click here for file
