# Supplementary material for: Continuity of midwifery care and gestational weight gain in obese women: a randomised controlled trial
Source: BMC Public Health. 2011 Mar 22;11:174. doi: 10.1186/1471-2458-11-174 (PMC3074543; doi:10.1186/1471-2458-11-174)
Supplement: Additional file 1 — CONSORT flow diagram. This flow diagram depicts the adherence of this study to CONSORT requirements [file 1471-2458-11-174-S1.DOC]

**Additional file 1: CONSORT Flow Diagram**

**Allocation**

**Analysis**

**Q1**

Booking visit <17 weeks

**Enrollment**

**Q2**

36 weeks

Assessed for eligibility (n= )

Excluded (n= )

♦  Not meeting inclusion criteria (n= )

♦  Declined to participate (n= )

♦  Other reasons (n= )

Analysed (n= )
♦ Excluded from analysis (give reasons) (n= )

Lost to follow-up (give reasons) (n= )

Discontinued intervention (give reasons) (n= )

Allocated to intervention (n= )

♦ Received allocated intervention (n= )

♦ Did not receive allocated intervention (give reasons) (n= )

Lost to follow-up (give reasons) (n= )

Discontinued intervention (give reasons) (n= )

Allocated to intervention (n= )

♦ Received allocated intervention (n= )

♦ Did not receive allocated intervention (give reasons) (n= )

Analysed (n= )
♦ Excluded from analysis (give reasons) (n= )

Randomized (n= )
