# Supplementary material for: Continuity of midwifery care and gestational weight gain in obese women: a randomised controlled trial
Source: BMC Public Health. 2011 Mar 22;11:174. doi: 10.1186/1471-2458-11-174 (PMC3074543; doi:10.1186/1471-2458-11-174)

**What do I do if I am gaining too much weight:**

Pregnancy is not a time for strict dieting. However you do not need to eat for two! There are some simple choices you ca n make that will help you to limit the amount of additional energy and managing your weight gain in pregnancy please ask your midwife or doctor for a referral to a Dietitian.

**Limit the amount of fat you eat.**

Reduce your intake of snack foods such as biscuits, cakes, chips, crisps and chocolate.

Reduce the amount of fat or oil used in cooking.

Choose low fat or reduced fat diary foods such as milk, yoghurt and chesses. These products still have all the calcium you need for your bones.

Avoid eating cream or sour cream.

Trim all fat off your meat before cooking.

Remove skin from chicken.

Limit high fat take-away foods.

**Limit high sugar foods.**

Drink water not soft drink or cordial.

Limit sweetened soft drinks

Limit fruit juices to once a day as these are high in natural sugar.

Limit chocolate. Lollies and sweets.

Go easy on desserts.

Try to minimise snacking, but if you do need to snack. Choose options such as fresh fruit, low fat yoghurt, dry biscuits with reduced fat hard cheese.

Try to do as much exercise as you can. Regular exercise can help prevent excess weight gain.

References:

Further information on pregnancy can be found:

Royal Women’s Hospital. http://www.thewomens.org.au/

Queensland Health Dieticians http://www.health.qld.gov.au/masters/copyright.asp

Institute of Medicine Revised Guidelines 2009 can be found at: http://iom.edu/obect.File/

Master/68/230.Report%20Brief%20-%20Weight%20Gain20During%20Pregnancy.pdf

This brochure has been reproduced and adapted by [recruitment site] with the kind permission of GV Health.

GV Health /Publication GVH0333

Prepared by the Nutrition & Dietetics Department—For review 2013

**Additional file 2: Informational leaflet**

**Pregnancy Weight Matters**

**Managing your weight gain during pregnancy.**

During pregnancy it is normal to gain w weight as your baby grows and your b body adapts to being pregnant.

H However, gaining too much weight or b being overweight can cause m complications for both you and your b baby. These complications can occur w while you are pregnant, during labour o or after your baby is born.

T The purpose of this information is to h help you to understand the risk of n gaining too much weight or being v overweight in pregnancy (to you and y your baby) and help you manage your w weight gain.

Weight is often a sensitive issue for women. In pregnancy many women struggle with body shape changes and you may feel uncomfortable discussing weight issues with your local health professional.

The following information will help you to understand why additional precautions are taken during outcomes for you and your baby.


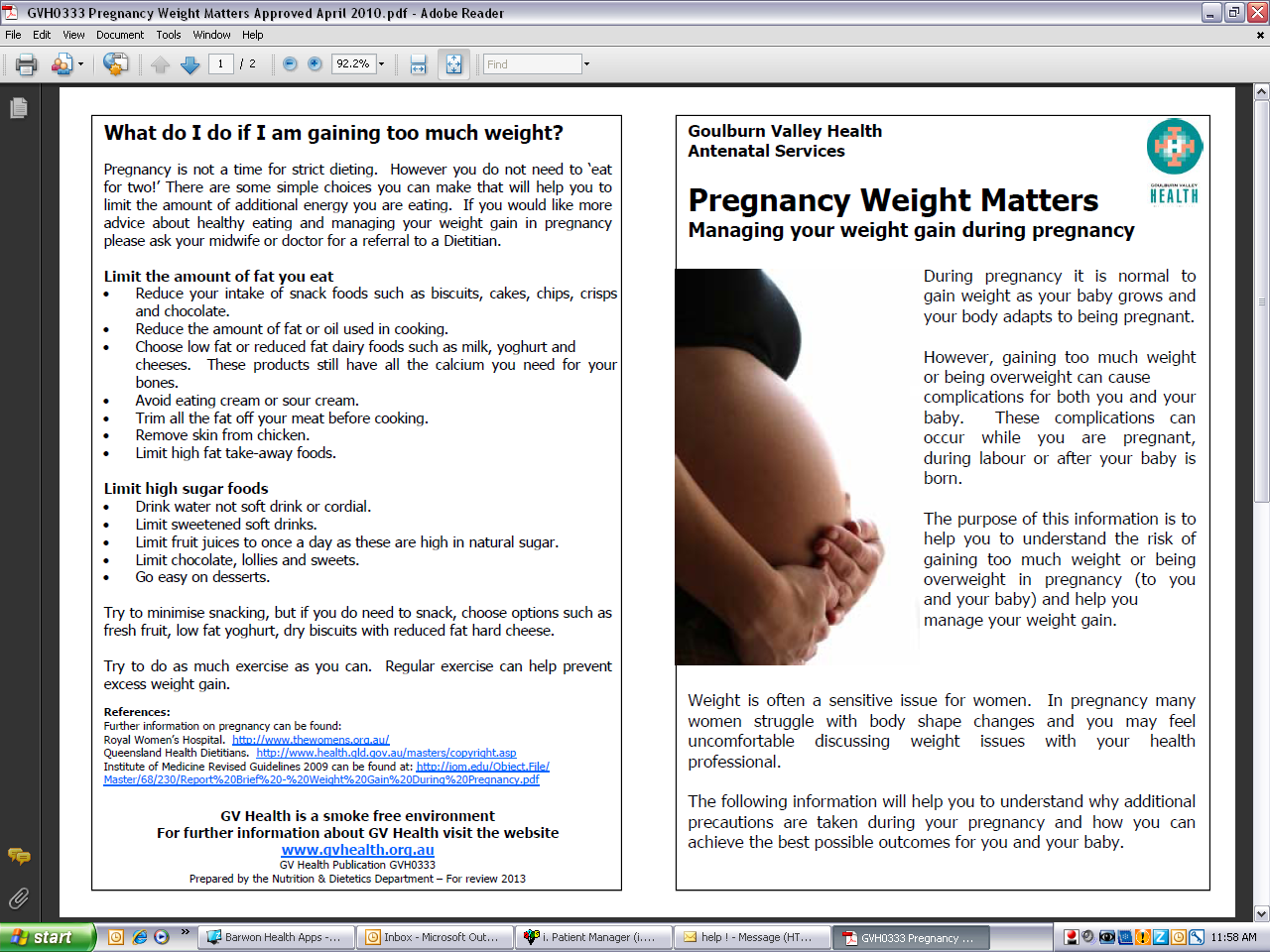

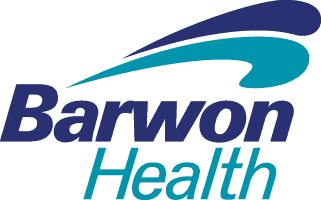


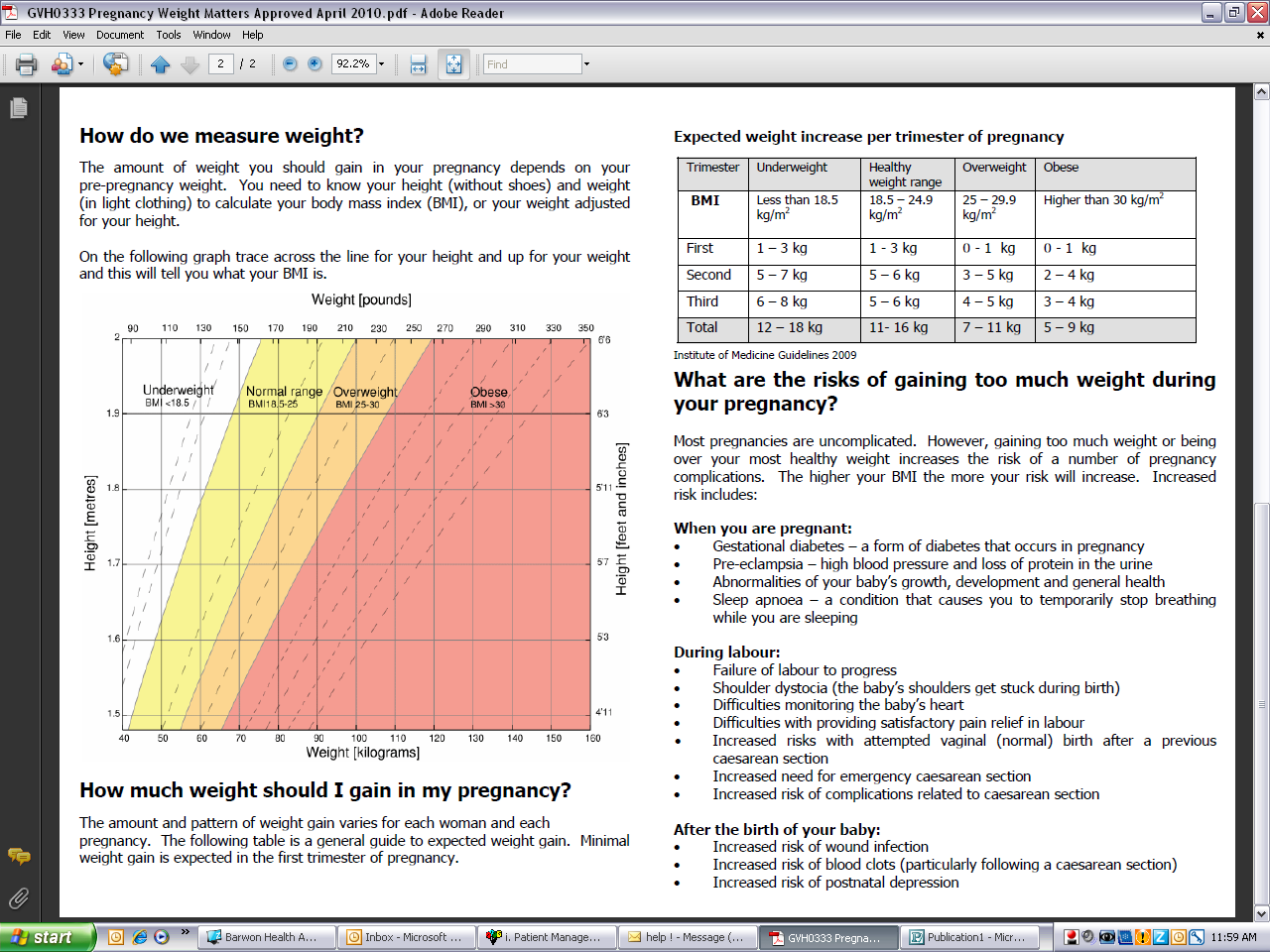

Supplement: Additional file 2 — Informational leaflet. This is the information leaflet on healthy weight gain that will be provided to women in both aims of this study. [file 1471-2458-11-174-S2.DOC]
